# Necrosis of Hard Palate

**Published:** 1898-03

**Authors:** H. C. Gilchrist

**Affiliations:** Nyack, N. Y.


AMERICAN JOURNAL

—OF—

DENTAL SCIENCE.

VOL. XXXI. BALTIMORE, MARCH, 1898. NO. 11.

ARTICLE I.

### NECROSIS OF HARD PALATE.

BY DR. H. C. GILCHRIST, NYACK, N. Y.

Read before Second District Dental Society, Newburg, N. Y., October 11, 1897.

We often meet peculiar and interesting cases in our practice which are worth recording, as they may be the means of helping others in the future if they should meet similar cases.

One day during the summer of 1893, there came to my office a young lady about twenty-three years of age, to consult me about a large swelling in the roof of the mouth, extending from the incisors to the soft palate, being one inch in thickness.

Upon getting the history of the case, I found that some time previously she had had a central incisor that had abscessed; her dentist having failed to destroy the abscess, she, in a fit of desperation, went to her family physician and had the tooth extracted.

A few days after the extraction the swelling appeared in the roof of the mouth; her physician after repeatedly lancing, finally told her that it was only a lump of flesh, and that it would "disappear after a while"; such was the condition when she came to me for advice.

Upon examination I found that the abscess sac was filled with pus, although she said her physician had lanced it that morning. I found he had lanced at the highest instead of at the lowest point, and consequently had not evacuated the sac. I then inserted my lance in the bottom of the sac, whereupon it discharged about half an ounce of thick yellow pus, which gave her immediate relief, much to her astonishment, as her doctor told her that there was nothing in it.

In a few days she returned with the swelling as large as ever. Again I evacuated it. This time I found that it was distinctly bone pus. I then came to the conclusion that there was necrosed bone, whereupon I took a small piece of broom straw, having first thoroughly soaked it in carbolic acid, then coating it with vaseline, I inserted it in the opening made with my lance and tied it fast to one o the incisors. This kept the abscess open and allowed the pus to discharge.

This I kept in the pus sac for two weeks, when I found that a fistulous opening had occurred at the junction of the soft and hard palate, and upon passing my probe through the opening, I found a peculiar roughness that led me to believe that there was a piece of dead bone trying to exfoliate, whereupon I gave the patient a small syringe and some hypo-sulphite of soda, with which I directed her to syringe out the pus sac twice a day. This treatment was continued for three weeks, reducing the sac very much in size, when upon examination I found that the necrosed bone had detached itself. I placed the patient under the influence of nitrous oxide, and opened the roof of the mouth from the central incisor, following the median line, to the beginning of the soft palate, having my assistant at hand with small sponges to care for the hemorrhage and prevent blood from getting in the air passages.

After turning back the flaps, I found the piece of necrosed bone which I removed. It was a piece of the superior maxillary from the left side—one half of an inch in length by one-sixteenth of an inch in thickness. After washing out the cavity with bi chloride of mercury (1-10,-000) I drew the edges of the flaps together and placed in four ligatures of waxed floss silk, which had been soaked in bi-chloride of mercury. I then placed in a small piece of broom straw again, to act as a drainage tube. In a week's time, the parts being united, I removed the ligatures. The next week I found that there was no discharge, and then removed the drainage and discharged the patient.

About a month after I saw the patient, and the mouth was thoroughly healed and in a perfect condition.—*Items of Interest.*

